# An Object-Independent ENZ Metamaterial-Based Wideband Electromagnetic Cloak

**DOI:** 10.1038/srep33624

**Published:** 2016-09-16

**Authors:** Sikder Sunbeam Islam, Mohammad Rashed Iqbal Faruque, Mohammad Tariqul Islam

**Affiliations:** 1 Space Science Centre, Institute of Climate Change, Universiti Kebangsaan Malaysia, Bangi 43600, Malaysia; 2 Dept. of Electrical, Electronic and Systems Engineering, Faculty of Engineering and Built Environment, Universiti Kebangsaan Malaysia, 43600 UKM, Bangi, Selangor, Malaysia

## Abstract

A new, metamaterial-based electromagnetic cloaking operation is proposed in this study. The metamaterial exhibits a sharp transmittance in the C-band of the microwave spectrum with negative effective property of permittivity at that frequency. Two metal arms were placed on an FR-4 substrate to construct a double-split-square shape structure. The size of the resonator was maintained to achieve the effective medium property of the metamaterial. Full wave numerical simulation was performed to extract the reflection and transmission coefficients for the unit cell. Later on, a single layer square-shaped cloak was designed using the proposed metamaterial unit cell. The cloak hides a metal cylinder electromagnetically, where the material exhibits epsilon-near-zero (ENZ) property. Cloaking operation was demonstrated adopting the scattering-reduction technique. The measured result was provided to validate the characteristics of the metamaterial and the cloak. Some object size- and shape-based analyses were performed with the cloak, and a common cloaking region was revealed over more than 900 MHz in the C-band for the different objects.

Currently, the application of metamaterials to invisibility cloaking operations has received significant interest from the scientific community. Metamaterial is a type of artificially constructed composite material that may have some exotic electromagnetic properties. These properties are different from those of naturally available materials. After the first exploration of metamaterial^1^, different design types were proposed in the literature for different applications^2^,^3^,^4^,^5^. Typically, periodic unit cells are adopted to form a bulk metamaterial, but the unit cell itself can be utilized in several applications, such as antenna design, specific absorption reduction (SAR), filter design, and cloak design^6^,^7^,^8^,^9^,^10^. Metamaterials may play a significant role in electromagnetic cloak design. Important applications of cloak include stealth coating of aircraft or missiles, especially for the defense sector of a country. Moreover, concealing small satellite from hostile radar is another important application of small cloak. An electromagnetic cloak can hide (cloak) an object electromagnetically. Various techniques have been followed by the researchers for cloaking operations, for example, geometry optics, transformation optics, and scattering reduction^11^,^12^,^13^,^14^,^15^. In the transformation optics (TO) method, a virtual ‘no-field’ region is created where the electromagnetic waves are guided in such a way to make the region undetectable. In the scattering reduction method, to hide an object electromagnetically, the scattering of electromagnetic waves from the object in any direction is reduced or prevented. Scattering reduction was also achieved for the TO-method^13^. This cloaking technique was applied for various practical applications at microwave^12^ and optical^16^ frequencies, including sensing and imaging purposes^17^,^18^.

This reduction of scattering can be achieved by adopting a homogeneous metamaterial with a low effective permittivity^19^. A properly designed metamaterial with a low or negative effective permittivity induces an opposite dipole moment between the core and the metamaterial shell to cloak an object. This cloaking technique is denoted as plasmonic cloaking. A good cloak reduces the normalized scattering cross section (NSCS) of the object to less than one. The normalized scattering cross section is the normalized cross section of the scattered energy of the cloaked object to scattered energy of the bare object. It was previously reported^20^ that a plasmonic cloak has the capacity to reduce not only dipolar scattering from small objects but also multi-polar scattering from large objects. Moreover, because the plasmonic cloak is not resonance dependent, it has robust behavior against ohmic absorption losses in the cloak^19^. Metamaterials with the epsilon-near-zero (ENZ) property have good prospects for single-layer cloak design^21^. Materials with the ENZ property have slow phase variation for a long distance that provides uniform phase distribution in a medium. Therefore, directive radiation can be obtained using such materials. Plasmonic cloak can be realized by the epsilon-near-zero material as well^22^.

Single-layer metamaterial-based plasmonic cloaks have been proposed in the literature, but most of these cloaks were cylindrical in shape and were not designed for C-band (4–8 GHz) operation. Moreover, they did not use ENZ metamaterial for the cloaking operation. For example, Xiaohui Wang *et al*.^23^, proposed a cylindrical cloak comprising a dielectric resonator-based metamaterial. Their cloak reduced the total scattering cross-section to less than one and operated in the X-band (8–12 GHz). D. Rainwater *et al*.^24^, presented a metamaterial-based 3D plasmonic cylindrical cloak. They suppressed the scattering of a finite length object in the S-band. Another metamaterial-based S-band (2–4 GHz) cloak demonstrated the ability to act as a cylindrical cloak^25^. Matekovits *et al*. produced a metasurface-based, single-layer cylindrical cloak that was designed with a width-modulated unit cell^26^. This cloak operated in the K-band (18–26.5 GHz) only. Recently, a metamaterial-based, single-layer rectangular cloak was designed for C-band operation^27^, where the near-zero refractive index property of the metamaterial was utilized for the cloaking operation, but the ENZ property was not used. Cloaking can be obtained using mu-near-zero property as well where effective permeability remains between zero and one. F. Bilotti *et al*. in ref. 28 proposed an ENZ material and MNZ (mu-near-zero) metamaterial-based electromagnetic cylindrical cloak for TE (transverse electric) and TM (transverse magnetic) polarization but they did not provide any experimental validation.

In this study, a new double-split-square-shaped metamaterial with a good effective medium ratio with the ENZ property is proposed for electromagnetic cloaking in the microwave region. The metamaterial demonstrates the ENZ property in the region of the C-band. The metamaterial was used to design a square-shaped, single-layer cloak as the cloak shell. The cloak was operating in the same band. Few shape- and size-oriented analyses were done to optimize the cloaking operation.

## Materials and Methods

**Figure 1:**
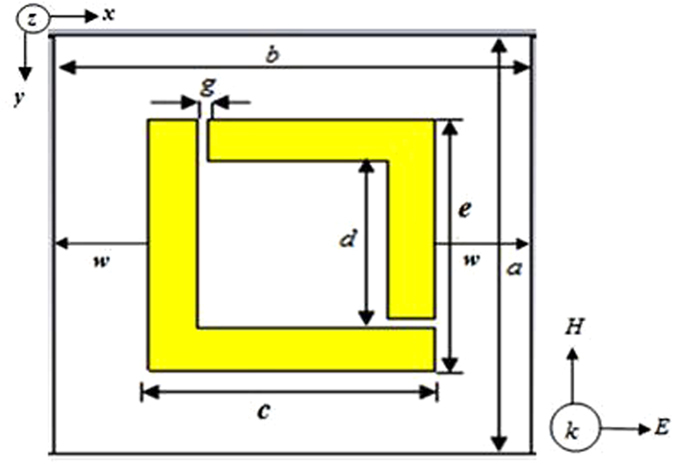
The schematic view of the unit cell.

The design of a metamaterial-based cloak starts with the design of a metamaterial. The structure and design specifications of the proposed metamaterial unit cell are displayed in Fig. 1. In this design, a square copper patch was split at the two corners to form a double-split-square-shaped structure. The structure was designed on a 1.6 *mm* thick FR-4 substrate material with dielectric property of ε = 4.3 + 0.02i. The length of each arm of the structure was engineered for a good effective medium ratio. The effective medium theory is the key to designing a proper metamaterial structure. For a good metamaterial, the wavelength of the applied field in the centimeter range of the unit cell should be kept in the millimeter range. However, according to the resonance frequency, the proposed metamaterial maintains the effective medium ratio approximately λ/a ≈ 6, where λ is the wavelength, and a = b = 10 *mm* is the length of the unit cell. The length of each of the arms and the split region in the proposed structure is accountable for generating consecutive inductive and capacitive effects. The rest of the design parameters of the unit cell are *c* = *6* *mm, d* = *3.8* *mm, g* = *0.2* *mm* and *e* = *6* *mm*. A *w* = 2 *mm* gap was maintained at each side from the resonator arms to the end of the substrate.

Simulation tool CST microwave studio was used for the numerical investigation of the proposed unit cell. Using the CST microwave studio, the transverse electromagnetic wave was propagated through the unit cell, placing the structure between two waveguide ports. After obtaining the S-parameters from the simulator, the method mentioned in ref. 29 was adopted to characterize the metamaterial.

## Results

**Figure 2:**
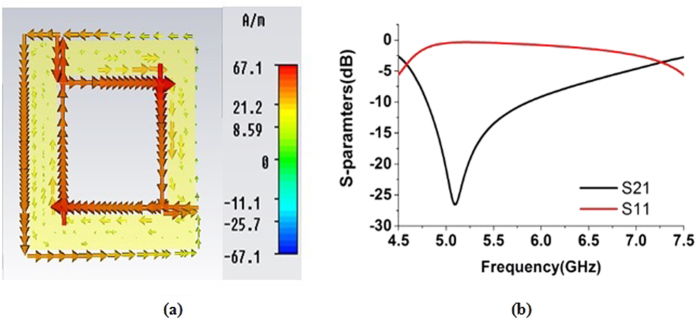
(**a**) Current distribution in the unit cell at 5.10 GHz. (**b**) S-parameters in dB for the unit cell.

At the frequency of 5.10 GHz, the current distribution in the unit cell is displayed in Fig. 2a. Current flow in the opposite direction is observed in each arm of the unit cell due to sustain the electrostatic condition caused by the skin effect on the metal stripe. If these two currents are equal, the fields cancel internally and the stop band occurs. The S-parameters magnitudes, including the transmission coefficient (S_21_) and the reflection coefficient (S_11_), are presented in Fig. 2b. Clear resonance is seen in the figure at the frequency of 5.10 GHz.

**Figure 3:**
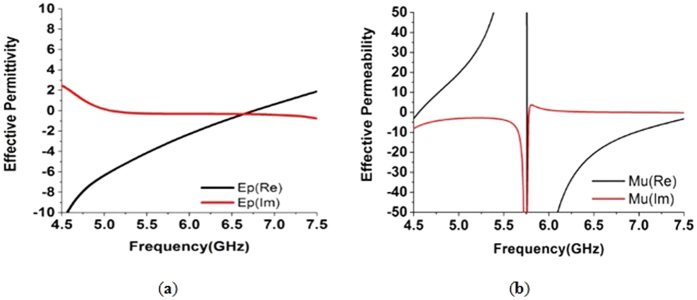
(**a**) Effective permittivity for the unit cell. (**b**) Effective permeability for the unit cell.

Figure 3a depicts the real and imaginary values of permittivity for the unit cell. The negative real peak for permittivity is found between frequency of 4.5 GHz to 6.71 GHz and a positive peak from 6.73 GHz to 7.5 GHz. However, this curve also has a near-zero region between the frequency of 6.34 GHz and 7.13 GHz. The imaginary part of the permittivity curve has a near-zero peak from 4.76 GHz to 7.59 GHz. The epsilon-near-zero region has appealing applications in the field of antenna and cloak design. This property can be applied in designing a single-layer cloak because materials with this feature create uniform phase distribution over a longer distance. In Fig. 3b, the real and imaginary peaks of the effective permeability are presented for the unit cell. The real peak of the permeability curve has negative magnitude from 5.76 GHz to 7.5 GHz and is positive before this range. The negative peak of the permeability curve occurs at higher frequency because in the higher frequency region, the current cannot cope up with applied field phase. However, the imaginary part of this curve has a near zero peak from 4.5 GHz to 7.5 GHz as well.

### Development of a Square-shaped cloak

The designed metamaterial was used to build a rectangular cloak. The scattering reduction method was adopted to design the cloak. To cloak an object according to the scattering reduction technique, a type of metamaterial shell encircled around the object to hinder the scattering from the object and to restore path of electromagnetic waves to its origin.

For designing a square-shaped cloak, it is an important issue to estimate the number of metamaterial unit cells according to the object to be cloaked. In this study, initial aim is to cloak a cylindrical object by a square-shaped cloak. Suppose, to cloak a cylinder of outer radius ‘*r*’, minimum ‘*n*’ number of unit cell is required to build a square-shaped cloak. For cloaking, the main target is to reconstruct the phase front of the incident electromagnetic (EM) waves behind the object to be cloaked. To reinstate the phase front of EM wave at the end of the cloak after passing the object, the EM wave have to spent the equal time inside the cloak as it takes to pass an object in the free space. If the side length of each wall of the square-shaped cloak is ‘*l*’, then from^30^,


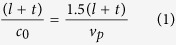


where, *v*_*p*_ = phase velocity, *c*_*0*_ = light speed. So, from Equation (1), inside cloak, *v*_*p*_ = 1.5*c*_*0*_.

**Figure 4:**
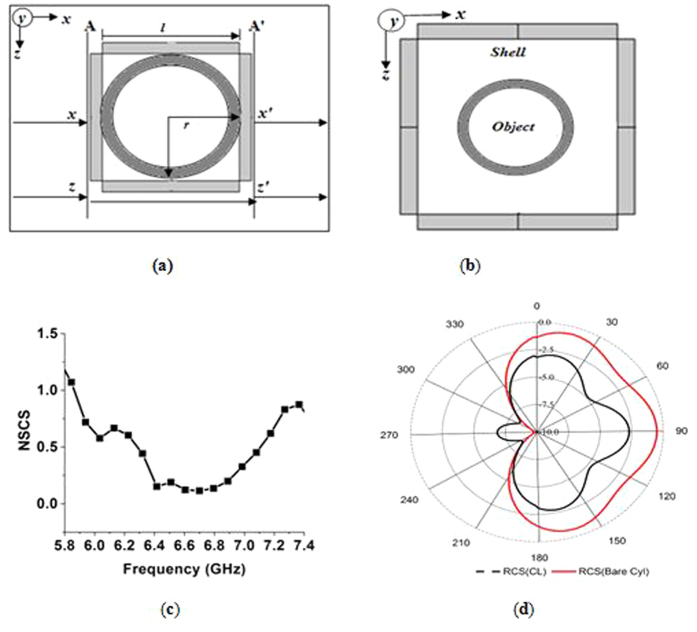
(**a**) Wave propagation at side incidence into the Square-cloak with cylindrical object (**b**) the cylindrical object inside the Square-cloak (**c**) Normalized scattering cross section (NSCS) of cloaked object normalized to the bare object (**d**) RCS pattern comparison of cloaked and bare metal object.

Similarly, according to the Fig. 4a, for a cylindrical object of outer radius *r*, if the EM waves strike at A and Z plane of the cloak, to reconstruct the wave front at A’ and Z’, the waves have to spent the same time as needed for the object in free space. Therefore,


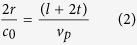


where, t = thickness of the substrate. Thus, *l* =  *3r* − *2t*

However, the length of the cloak wall contains *n* number of unit cells. So,


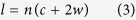


Therefore, from Equations (1), (2) and (3) the minimum number of unit cell needed is,


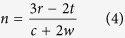


As a result to cloak a cylinder of outer radius *r* = *15* *mm*, according to Equation (4) minimum *n* = *4.18* or *n* ≈ *4* number of unit cell required at each side of the cloak and for *r* = *9* *mm*, minimum n = *2.3* or *n* ≈ *2* number of unit cell required at one side of the cloak. Similarly, for a cylinder of outer radius *r* = *5* *mm,* minimum *n* ≈ *1,* number of unit cell needed to cloak. Moreover, additional top up can be made as well according to the height of the cylinder. Similarly, for edge incidence of waves required number of unit cell is,


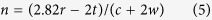


According to Equation (5), for *r* = *9* *mm*, minimum n = *2.2* or *n* ≈ *2* number of unit cell required at every side of the cloak and for *r* = *5* *mm,* minimum *n* ≈ *1,* number of unit cell needed to cloak. In this study, two unit cells were taken at each side of the wall of the square-cloak, because it makes the measurement process easy with waveguides for cloaking a copper cylinder with an outer and inner radius of 9 *mm* and 8 *mm* respectively. Therefore, total 8 unit cells were needed to form the cloak at the base row for all four walls of the square-cloak. The height of the cylinder was 20 *mm*. So, according to the height of the object, another row of unit cells was added to the top of the base line of the cloak.

Therefore, four 20 × 20 *mm*^2^ dielectric walls were used as the cloak shell. Each of the walls contained four unit cells of the proposed metamaterial. The cylindrical object was placed inside the cloak. The position of the cylinder was maintained so that the distance between the center of the cylinder and any of the walls was the same. The height of the metal cylinder was equal to the height of the cloak. In Fig. 4b, the object within the cloak shell is displayed. Performance comparison between the cloaked and uncloaked object was investigated to evaluate the cloaking effect. For the numerical investigation of the cloaking performance, the uncloaked object was first excited by the plane electromagnetic waves and then the cloaked object was analyzed in the same way. For both of the cloaked and uncloaked cases, the electromagnetic wave was propagated through the *x*-axis. To verify the qualitative performance of the cloak, the normalized scattering cross section (NSCS) was calculated. The total scattering cross section (TSCS) of an object was defined by the ratio of the scattered and incident energy of the object. To determine the NSCS of an object, the total scattering cross section of the cloaked object was normalized by the total scattering cross section of the uncloaked object. For the evaluation of the scattering cross section, the previously published equation^22^ was adopted.

In Fig. 4c, the simulation result of the NSCS of the cloaked object normalized to the uncloaked object is shown for the *xz*-*plane*. Figure 4c shows that at 6.70 GHz, the NSCS curve has the lowest value close to zero of 0.11, which indicates the optimum cloaking operation in the range. At 6.70 GHz, the effective permittivity of the metamaterial was ε = −0.087-j0.344 which is considered as ENZ value. Opposite dipole moment created by the ENZ region caused this lowest value of NSCS. Here the principal effect responsible for scattering reduction is the flat phase front of the scattered field that do not deform the incident waves. Moreover, an ENZ shell based cloak marginally relies on permittivity of object inside as the electric field is expelled out of the cloaked object. In square ENZ shell, on the outer surface of the cloaked object, the typical element of the electric induction ought to be persistent. In case of ENZ region of the material, the permittivity inside the shell is equivalent to almost zero. Since the permittivity inside the shell is equivalent to zero, the electric induction and its typical elements are equivalent to zero. The values of permittivity inside the cloaked object have definite values, though the normal component of the electric field inside the cloaked object has to be equivalent to zero. Therefore, all power lines of the electric field are brought to the volume of the cloaked object. After being scattered form the object, the scattering components rush towards the shell and are neutralized by local negative polarizability. However, the NSCS is below one in wide ranges of the frequency from 5.93 GHz to 7.27 GHz. Therefore, these frequency ranges can also be considered as part of the cloaking zone for the object. In Fig. 4d, the comparison between scattering patterns of the uncloaked and cloaked object are provided, and clear scattering suppression is visible for the cloaked object.

**Figure 5:**
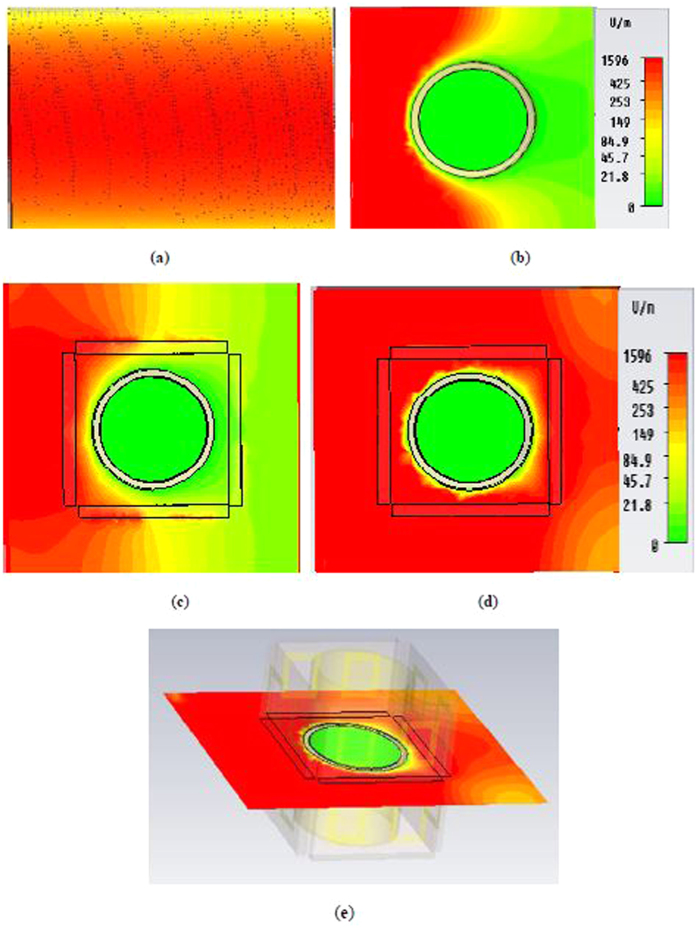
Distribution of E-field in the *xz-plane* for, (**a**) free space; (**b**) uncloaked object; (**c**) object at uncloaked frequency inside the cloak shell; (**d**) object at cloaked frequency (6.70 GHz) inside the cloak shell (**e**) transparent side view of the cloaked object at 6.70 GHz.

Figure 5 depicts the E-field map in the *xz-plane* for the open environment, an uncloaked object, the object inside the cloak shell at a normal frequency, and the object inside the cloak shell at a frequency where the cloak operates. Compare to the E-field distribution in the free space in Fig. 5a, clear E-field distortion in the forward direction (i.e., shadow at the right side of the bare object) is apparent in Fig. 5b, which indicates scattering in the forward direction for the object.

In Fig. 5c, almost the same (i.e., bare object case) E-field distribution is visible for the object at the uncloaked frequency inside the cloak shell. However, in Fig. 5d, it is evident from the E-field map that at the cloaked frequency of 6.70 GHz, proper wave front restoration is observed. Therefore, in the cloaked frequency, the forward E-field distortion (i.e., shadow behind the object) is improved due to the good scattering cancellation capability of the adopted metamaterial shell. Here, metamaterial shell creates a dipole moment of opposite sign that brings the waves back to the original path.

**Figure 6:**
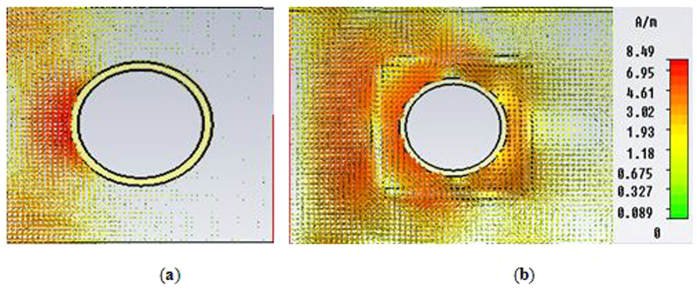
Distribution of H-field in the *xz-plane* for, (**a**) uncloaked object (**b**) cloaked object at cloaked frequency.

In Fig. 6, the H-field distribution in the *xz-plane* for the bare (in Fig. 6a) and cloaked (in Fig. 6b) object is presented. Similarly, to the E-field distribution, forward scattering was visible for the uncloaked object, and clear wave front reconstruction behind the object was seen for the cloaked object at the cloaked frequency.

### Experimental validation for the material and the cloak

**Figure 7:**
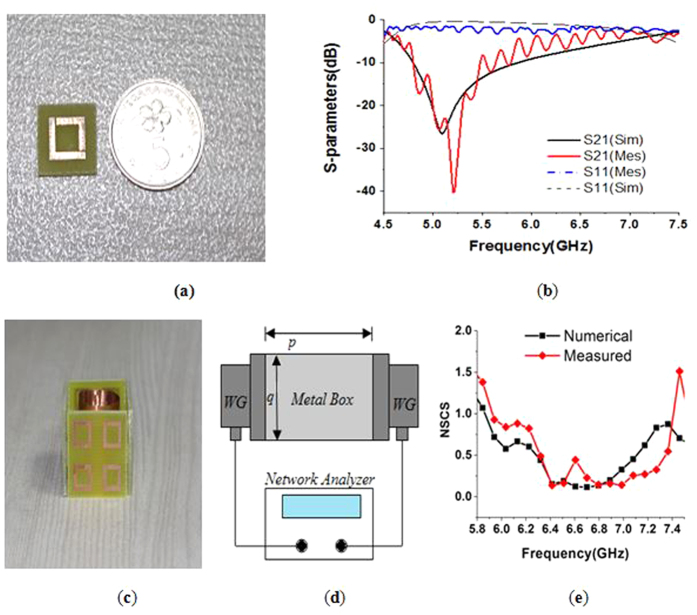
(**a**) Fabricated prototype of the material unit cell (**b**) comparison of the experimental and numerical transmittance of the unit cell (**c**) geometry of the cloak with the object (inside) (**d**) measurement setup for the cloak (**e**) comparison of the experimental and numerical NSCS of the cloaked object normalized to the bare object.

For the experimental validation, a basic unit cell of the material was fabricated. The fabricated unit cell is shown in Fig. 7a. Two rectangular-shaped, C-band WR137 wave-guides were adopted as the transmitter and receiver ports according to the simulation set up. The two wave-guides were connected to a vector network analyzer (N5227A). The fabricated prototype was placed between the transmitter and the receiver wave-guide, and the S-parameters were measured. The measured results are shown in Fig. 7b, where the experimental S-parameters are in good agreement with the simulated results. The measured transmittance was found at 5.18 GHz instead of at the simulated value of 5.10 GHz.

In Fig. 7c, the geometry of the proposed cloak is displayed with the metal object inside. For the purpose of the measurement, as with the structure of the simulation arrangement, the cloak structure was built, and the open space measurement method was adopted. The measurement setup is shown in Fig. 7d. A copper object of the same size was used for the experiment. The same two C-band WR137 wave-guides (WG) were used as the transmitter and receiver, and they were connected to the VNA. However, according to the size of the waveguide, an aluminum box (*q* = 49.4 *mm* high, 68.5 *mm* wide and *p* = 44 *mm* long) with two open sides was prepared. The two sides of the box were kept open so that the two wave-guides could be inserted facing each other in the box. The cloak structure was inserted inside the box between the waveguides. For the calculation of the normalized scattering width, a previously published method^13^ was followed. For the calculation of the normalized scattering width, both the scattered and incoming fields were measured. The incoming field (E_inc_) was measured without placing an object between the waveguides. Similarly, the scattered field (E_sca_) was measured by setting the object between the waveguides to provide the total field (E_t_), including the incoming and the scattering field, i.e., E_t_ = E_inc_ + E_sca_. By subtracting the E_inc_ from the E_t_, E_sca_ is calculated. Moreover, field measurement with the object within the cloak was also performed. The NSCS of the cloaked object normalized to the bare object was then calculated. In Fig. 7e, the experimental NSCS of the cloaked object normalized to the bare object was compared with the numerical NSCS of the cloaked object normalized to the bare object. From Fig. 7e, it is evident that the experimental curve shifted to the higher frequency side and had the lowest width at 6.79 GHz instead of 6.70 GHz, with a value of 0.14. There are several potential reasons for the shift. Fabrication error in the cloak shell or noise in the open space measurement process can result in the shift. Conversely, error between the inter-shell gaps may cause significant shifting. Proper positioning of the object inside the cloak is another important issue that might cause this shifting in the higher frequency side. In addition, at 6.79 GHz, the effective permittivity of the material unit cell was ε =  0.10-j0.35. However, the experimental NSCS curve has a value below one in a wide range of frequencies from 5.96 GHz to 7.38 GHz. Therefore, these frequency ranges can be considered as the practical cloaking zone for the object as well.

## Discussion

### Effect of the object shape in the cloak

**Figure 8:**
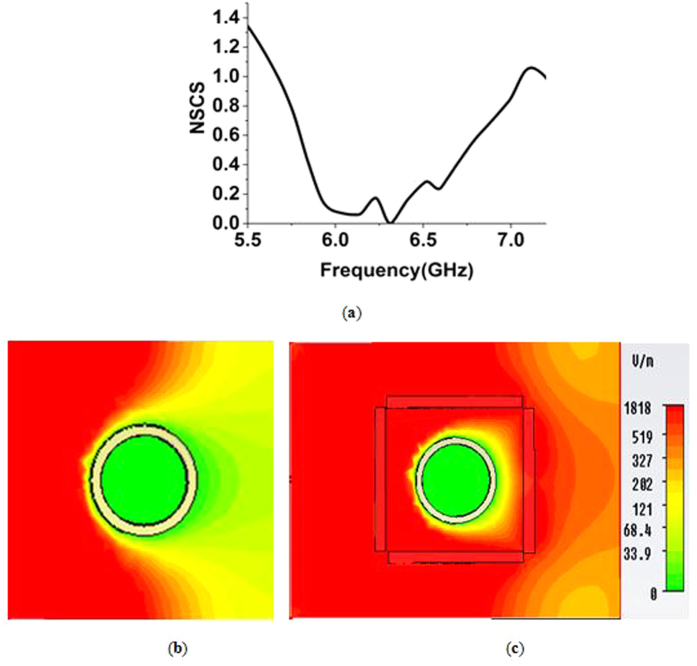
(**a**) NSCS of the cloaked object (shortened shape) normalized to the bare object (**b**) distribution of E-field in the *xz-plane* for, uncloaked object (shortened shape) (**c**) E-field distribution for the cloaked object with shortened shape at the cloaked frequency of 6.35 GHz.

For further investigation, the object shape was changed, and the effect on the cloaking performance was observed. The inner and the outer radius of the metal object were shortened to 4 *mm* and 5 *mm*, respectively. Figure 8a shows the NSCS of the cloaked object normalized to the bare object for the shortened object.

Figure 8a shows that the lowest peak of the NSCS curve shifted to the left side (*i.e.,* lower frequency side) at 6.35 GHz instead of at 6.70 GHz, with a value of 0.03. At this point, the effective permittivity is ε = −0.94-j0.32. However, the effect of ENZ property is the reason for cloaking operation here. However, the curve also has values less than one in the wide range of frequency from 5.75 GHz to 6.95 GHz. Figure 8b,c depict the electric field distribution in the *xz*-plane for the uncloaked and cloaked shortened object, respectively. Compared to the uncloaked object in Fig. 8b, the cloaked object in Fig. 8c shows clear wave front reconstruction in the forward direction.

### Effect of a square-shaped object in the cloak

**Figure 9:**
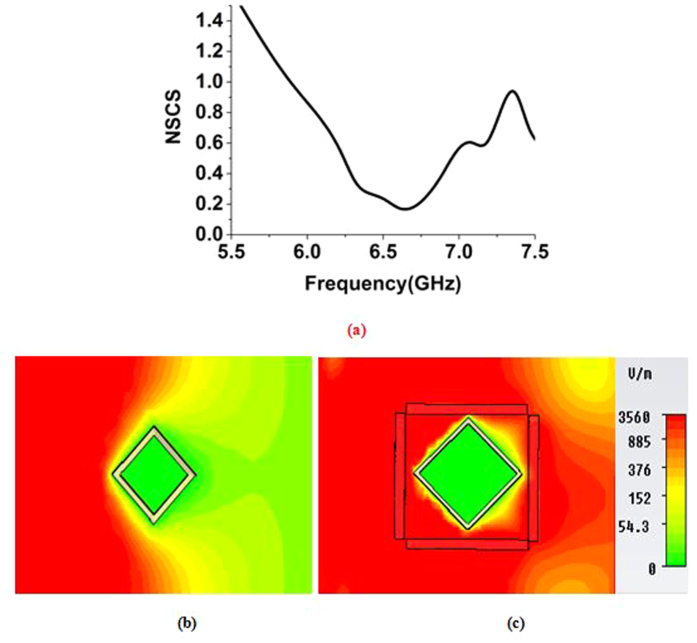
(**a**) NSCS of the cloaked square-shaped object normalized to the bare object (**b**) distribution of E-field in the *xz-plane* for, uncloaked square-shaped object (**c**) E-field distribution for the cloaked square-shaped object at the cloaked frequency of 6.63 GHz.

A square-shaped copper object was chosen to further analyze the effect of the cloak. The height of the square-shaped object was equal to the height of the cloak, and the length of the square was chosen in such a way that can be placed equally instead of a 9 *mm* radius cylinder. Figure 9a shows the similar NSCS graph for the square-shaped object.

From Fig. 9a, it is visible that the minimum peaks in the normalized scattering width curve for the square-shaped object is at 6.63 GHz in the C-band, with values of 0.16. The effective permittivity for the metamaterial at these points of frequency was ε = −0.35-j0.33. It is evident that, these low values of normalized scattering cross section were achieved for the opposite dipole moment created by the shell due to near zero negative permittivity effect. However, values less than one were also seen from 5.94 GHz to 7.36 GHz, which can also be considered as part of the cloaking zone. In Fig. 9b, the electric field distribution for the uncloaked square-shaped object is shown, and the forward field distortion is clear. Similarly, in Fig. 9c, the electric field distribution for the cloaked square-shaped object in the cloaked frequency is shown, and the distortion of the field in the forward direction was attenuated, which indicates that the wave is bending back to its original path due to the cloak.

### Effect of a pentagon-shaped object in the cloak

**Figure 10:**
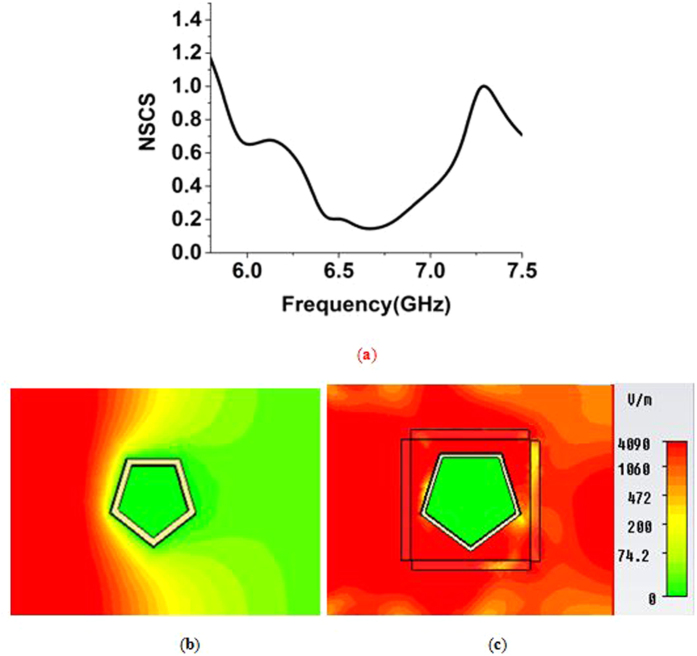
(**a**) NSCS of the cloaked pentagon-shaped object normalized to the bare object (**b**) distribution of E-field in the *xz-plane* for, uncloaked pentagon-shaped object (**c**) E-field distribution for the cloaked pentagon-shaped object at the cloaked frequency of 6.65 GHz.

The effect of a pentagon-shaped copper object was investigated as well to see the cloaking performance. The height of the object was equal to the cloak height. The shape was chosen in such a way so that it can be placed instead of a cylinder with an outer radius 9 *mm*. Figure 10a shows the similar NSCS curve for the pentagon-shaped object.

Figure 10a shows that the normalized scattering width curve has the lowest values at 6.65 GHz, with values 0.14. The lowest NSCS point of frequency for pentagon-shaped object almost matches the point obtained for square-shaped one. It also follows the NSCS curve of Fig. 4c. The effective permittivity for the metamaterial at this points of frequency was ε = −0.27-j0.33. Like the square-object, almost similar effect of ENZ property is visible here for the same size pentagon-object. The curve also exhibits values less than one from 5.94 GHz to 7.27 GHz. This range of frequency can also be utilized as region of the cloaking zone. In Fig. 10b, the electric field distribution for the uncloaked pentagon-shaped object is shown, and forward field distortion is visible. Similarly, in Fig. 10c, the distribution of the electric field for the cloaked pentagon-shaped object in the cloaked frequency is shown, and wave front reconstruction in the forward direction is visible, which indicates scattering reduction in the forward direction (right side of the object), and the electromagnetic waves return to their original path due to the cloak.

## Conclusions

In conclusion, a new double-split-square-shaped metamaterial was introduced in this study and was used in the cloaking operation. The proposed metamaterial-based cloak produced approximately zero scattering cross section for a cylindrical object, where the metamaterial shows ENZ property. Moreover, the operations of the metamaterial and cloak were experimentally analyzed. Shape-based analyses were performed with a shortened cylinder and same size square-shaped and pentagon-shaped object and thereafter the cloaking performances were recorded. It was observed that, for the object of equal size, the cloak operates in the ENZ region of the metamaterial. For all of these objects, the cloak demonstrated cloaking performance in the C-band. Moreover, for all of these different objects, the frequency range from 5.94 GHz to 6.95 GHz (more than 900 MHz) was a common range where the cloak provided values less than one for the normalized scattering cross section. Therefore, this range is the common cloaking region for all of these objects.

## Additional Information

**How to cite this article**: Islam, S. S. *et al*. An Object-Independent ENZ Metamaterial-Based Wideband Electromagnetic Cloak. *Sci. Rep.* **6**, 33624; doi: 10.1038/srep33624 (2016).
